# Holmium Laser Enucleation of the Prostate Vs. Bipolar Transurethral Resection of the Prostate for Small-Volume Prostates: A Comparative Analysis of Clinical Outcomes

**DOI:** 10.5152/tud.2026.25132

**Published:** 2026-03-13

**Authors:** Abdullah Gölbaşı, Burak Elmaağaç, Hüseyin Biçer, Ali Yasin Özercan, Sefa Günal, Murat Keske

**Affiliations:** 1 Department of Urology, University of Health Sciences Faculty of Medicine, Kayseri City Hospital, Kayseri, Türkiye; 2 Department of Urology, Kayseri City Hospital, Kayseri, Türkiye; 3 Kayseri Memorial Hospital, Kayseri, Türkiye

**Keywords:** Holmium, lasers, prostate, urinary tract

## Abstract

**Objective:**

Benign prostatic hyperplasia is a common cause of lower urinary tract symptoms (LUTS) in aging men. While transurethral resection of the prostate (TURP) is the traditional surgical standard, holmium laser enucleation of the prostate (HoLEP) has emerged as a safe and effective alternative. Evidence comparing both techniques in small prostates (<50 mL) remains limited.

**Methods:**

This retrospective study included male patients aged 40-80 years who underwent bipolar TURP (B-TURP) or HoLEP between 2023 and 2025 for LUTS refractory to medical therapy, with prostate volume <50 mL. Pre- and postoperative parameters, including operative time, resected tissue weight, irrigation volume, hospital stay, catheterization time, and functional scores (International Prostate Symptom Score (IPSS), International Index of Erectile Function-5 (IIEF-5), International Consultation on Incontinence Questionnaire-Short Form (ICIQ-SF), peak urinary flow rate (Qmax), post-void residual urine (PVR)) were compared.

**Results:**

No significant differences were found between groups regarding age, body mass index, comorbidities, prostate-specific antigen, prostate volume, ICIQ-SF scores, IIEF-5, complication rates, or postoperative hemoglobin decrease (all *P* > .05). Holmium laser enucleation of the prostate showed longer operative time, greater irrigation use, and higher resected tissue weight/percentage (*P* = .03, .02, <.001, <.001), while hospital stay and catheterization were shorter (both *P* < .001). Both procedures improved Qmax and IPSS and reduced PVR (all *P* < .001); the increase in Qmax was greater in HoLEP (*P* = .025).

**Conclusion:**

Both B-TURP and HoLEP are safe and effective for small prostates; however, HoLEP offers advantages in resected tissue weight, Qmax improvement, and shorter hospital stay/catheterization.

**Main Points**

- Holmium laser enucleation of the prostate (HoLEP) achieved greater tissue resection and higher peak urinary flow rate improvement.
- Catheterization and hospital stay were shorter after HoLEP.
- Both HoLEP and bipolar-transurethral resection of the prostate (B-TURP) were safe with similar complication rates.
- HoLEP showed superiority over B-TURP in several clinical aspects.

## Introduction

Benign prostatic hyperplasia (BPH) is a histopathological condition marked by the enlargement of prostate tissue. This enlargement can cause obstruction, leading to lower urinary tract symptoms (LUTS), particularly in older men. However, prostate volume does not directly predict the severity of LUTS, as prostates of varying sizes may produce similar symptom profiles.^1^^,^^2^

In the treatment of LUTS associated with BPH, surgical approaches are used when medical treatment is insufficient. Both endoscopic and open methods have been used in surgical treatment for many years. With technological advances, the diversification of endoscopic methods, particularly holmium laser enucleation of the prostate (HoLEP), has led to questioning the role of transurethral resection of the prostate (TURP), which has been considered the reference technique in endoscopic treatments for many years.^3^^,^^4^

Patient-specific evaluation is essential to determine which individuals are most likely to benefit from a particular surgical technique. Holmium laser enucleation of the prostate has been recognized as a standard and effective treatment option for BPH, providing outcomes comparable to those of open prostatectomy, particularly in patients with large prostate volumes. Both approaches have been shown to achieve similar improvements in LUTS.^5^^-^^8^

However, compared to TURP, which is considered the standard surgical method for symptomatic small prostate volumes, fewer studies have evaluated the efficacy of HoLEP in this specific patient group. Most existing HoLEP studies have predominantly focused on large prostate volumes, leaving a relative lack of direct comparative data for small prostates. Moreover, head-to-head comparisons between HoLEP and bipolar TURP (B-TURP) in prostates <50 cc remain limited. Therefore, this study was designed to comparatively evaluate the safety and efficacy of B-TURP and HoLEP in small prostate volumes (<50 cc), aiming to address this gap and contribute additional evidence to the existing literature.

## Material and Methods

### Patient Selection and Data Collection

This retrospective, cross-sectional, comparative study included patients aged 40-80 years who underwent B-TURP or HoLEP between 2023 and 2025 due to LUTS unresponsive to medical therapy and had a prostate volume of <50 mL. Prostate volume was evaluated using ultrasound or magnetic resonance imaging and calculated with the ellipsoid formula [0.52 × transverse × anteroposterior × craniocaudal diameters (cm^3^)]. Patients with prostate cancer, a history of neurogenic bladder, those who could not discontinue anticoagulant therapy, or those who had previously undergone BPH surgery were excluded. Data were collected from patient files and the hospital’s electronic health record system.

### Study Design and Data Analysis

Patients were divided into 2 groups according to the surgical procedure performed (B-TURP and HoLEP). A comparative analysis was performed to evaluate differences in demographic, clinical, and surgical outcomes between the groups. Demographic data included age, body mass index (BMI), prostate-specific antigen (PSA) levels, and preoperative and postoperative hemoglobin changes. Additional parameters, including prostate volume, operative time, irrigation fluid volume, resected tissue weight and percentage, and routine scoring systems (International Consultation on Incontinence Questionnaire-Short Form (ICIQ-SF), International Prostate Symptom Score (IPSS), International Index of Erectile Function-5 (IIEF-5)), peak urinary flow rate (Qmax), post-void residual urine (PVR), length of hospital stay, and catheterization duration, were compared. Functional outcomes and symptom scores were assessed at 6 weeks postoperatively, and hemoglobin levels were remeasured at 24 hours. Complications were recorded according to the Clavien–Dindo classification.^9^

### Surgical Procedure

All procedures were performed by 2 experienced surgeons under spinal anesthesia with the patients placed in the lithotomy position. A 26 Fr continuous-flow resectoscope with a 30° cystoscopic lens was used in both techniques, and normal saline served as the irrigation fluid.

In the B-TURP group, bipolar plasma loop resection (Plasma EDGE, Lamidey-Noury, France) was performed at 200 W/120 W cutting/coagulation settings.

In the HoLEP group, enucleation was carried out using a 150 W holmium: YAG laser (Quanta System, Saronno, Italy) with a 550-nm end-firing fiber, employing the 3-lobe technique (40 Hz/2.0 J for enucleation, 40 Hz/1.2 J for coagulation). Adenomas were subsequently morcellated and removed from the bladder using a mechanical tissue morcellator (Hawk, Minitech Co., Shenzhen, China) with indirect nephroscope guidance.

In both groups, resected tissue was immediately weighed and sent for histopathological evaluation. At the end of the procedures, a 22 Fr 3-way Foley catheter was inserted with continuous bladder irrigation and removed within 1-2 days postoperatively.

All surgeries were performed by 2 urologists who had completed the learning curve for both HoLEP and B-TURP procedures, each performing over 100 procedures of both techniques annually.

### Statistical Analysis

Sample size calculations were performed a priori using G*Power software (version 3.1, Universität Düsseldorf, Germany). The primary endpoint was the change in IPSS score at 6 weeks postoperatively. A large effect size (Cohen’s *d* = 0.8) was assumed based on previous studies.^10^ With a 2-sided significance level (*α* = 0.05) and 95% power. The calculation indicated that at least 49 patients per group (total 98 patients) were required.

Statistical analyses were performed using IBM SPSS Statistics, version 30 (IBM Corp., Armonk, NY, USA). The normality of continuous variables was assessed using the Kolmogorov–Smirnov test. Normally distributed data are presented as mean ± standard deviation, non-normally distributed data as median (interquartile range), and categorical data as number (percentage). Between-group comparisons of continuous variables were performed using the Student’s *t*-test for normally distributed variables and the Mann–Whitney *U*-test for non-normally distributed variables. Within-group pre- and postoperative comparisons of Qmax, IPSS, IIEF-5, PVR, and hemoglobin values were analyzed using the Wilcoxon signed-rank test. Categorical variables were compared using the chi-square test or Fisher’s exact test, as appropriate. A *P*-value <.05 was considered statistically significant.

## Results

### Comparison of Demographic, Perioperative, and Postoperative Characteristics Between the Groups

There were no significant differences between the groups regarding age, BMI, comorbidities, PSA, prostate volume, ICIQ-SF scores, or complication rates according to the Clavien–Dindo classification (all *P* > .05). However, compared to the B-TURP group, the HoLEP group demonstrated longer operation times, greater irrigation fluid usage, and higher resected tissue weight and percentage (*P* = .03, *P* = .02, *P* < .001, and *P* < .001, respectively). Conversely, hospital stay and catheterization duration were significantly shorter in the HoLEP group (both *P* < .001) (Table 1).

### Within-Group and Between-Group Comparisons of Clinical and Functional Outcomes

Comparison of pre- and postoperative functional outcomes demonstrated significant improvements in Qmax and IPSS, as well as a significant reduction in PVR, in both HoLEP and B-TURP groups (all *P* < .001). Among these outcomes, only the increase in Qmax was significantly greater in the HoLEP group compared to the B-TURP group (*P* = .025). IIEF-5 scores did not change significantly in either group (*P* = .393 and *P* = .195, respectively), and no intergroup difference was observed (*P* = .212).

Regarding clinical parameters, postoperative hemoglobin levels decreased significantly in both groups compared to preoperative values (both *P* < .001); however, the extent of hemoglobin reduction did not differ significantly between groups (*P* = .494) (Table 2).

## Discussion

In the present study, the preoperative and postoperative data as well as the clinical efficacy of B-TURP and HoLEP were comparatively evaluated in patients with prostate volumes <50 mL and LUTS. The analysis demonstrated that the HoLEP group achieved a higher weight and percentage of resected tissue compared to the B-TURP group, while hospital stay and catheterization duration were significantly shorter. Conversely, the HoLEP group was associated with longer operation times and greater irrigation fluid usage.

Functionally, both groups demonstrated significant postoperative improvements in Qmax and IPSS, accompanied by a marked reduction in PVR. In the intergroup comparison, only the increase in Qmax was significantly greater in the HoLEP group. Postoperatively, both groups exhibited an average hemoglobin decrease of approximately 1 g/dL at 24 hours, with no significant difference between them.

For many years, TURP has been regarded as the standard surgical treatment for small- to medium-sized prostates, whereas open prostatectomy has traditionally been the standard for large prostates in the management of BPH. Even today, TURP accounts for approximately 80% of endoscopic prostate surgeries.^4^ However, with advances in technology, HoLEP has become increasingly adopted by urologists. Holmium laser enucleation of the prostate is commonly considered a transurethral enucleation technique that can replace open prostatectomy for large prostate volumes, and most studies in the literature have primarily focused on this patient population.^5^^,^^3^

Herein, the efficacy of HoLEP and TURP in patients with small prostate volumes, which has been addressed in a limited number of studies in the literature, was evaluated using a comparative analysis method. According to the findings, both surgical techniques were found to be effective and safe treatment options for reducing LUTS. Similarly, in the matched analyses conducted by Magistro et al, both HoLEP and TURP were reported to provide significant improvement in LUTS, and both procedures were found to be safe.^11^^,^^12^

In the intergroup comparison, the improvement in Qmax was significantly greater in the HoLEP group. While this finding may be partly attributable to the higher weight and proportion of resected tissue in the HoLEP group compared to the B-TURP group, other contributing factors cannot be excluded. Previous studies have suggested that the relationship between the amount of tissue removed and the improvement in Qmax may be influenced by baseline detrusor function. This association appears to be relatively weak in patients with normal detrusor activity but more pronounced in those with detrusor hypoactivity. As no preoperative urodynamic evaluation was performed in the cohort, differences in bladder function or subtle variations in obstruction severity could not be fully assessed, and no subgroup analysis based on detrusor function could be conducted.^13^^,^^14^

In the study, the IIEF-5 and ICIQ-SF scoring systems were used to evaluate sexual function and urinary continence at the 6th postoperative week. No significant change in IIEF-5 scores was observed in either group, and none of the patients developed stress or urge incontinence during the postoperative period. Similarly, a meta-analysis published in 2022 reported that both HoLEP and TURP procedures had no significant adverse effects on erectile function.^15^

When evaluating operative times, it was observed that the HoLEP procedure required approximately 10 minutes longer on average. Although prostate volumes were small, it is believed that this difference originates from the morcellation phase of the enucleated tissues, which is an integral component of the HoLEP technique. Steps such as switching to a nephroscope during morcellation and waiting for the bladder to fill adequately to achieve optimal visualization may contribute to the prolonged operative time. A similar time difference has been reported in several studies in the literatüre.^11^^,^^16^

The longer duration of the HoLEP procedure also results in a greater amount of irrigation fluid being used simultaneously. In the analysis, the volume of saline utilized in the HoLEP group was higher than that in the B-TURP group. Previous studies have reported that increased irrigation volumes, particularly in cases involving large prostates, may lead to iso-osmolar fluid overload and complications requiring intensive care due to higher fluid absorption.^17^ However, since only patients with small prostate volumes were evaluated in the present study, the clinical relevance of this difference was not observed.

According to the findings, the HoLEP group experienced a shorter hospital stay and catheterization duration compared to the B-TURP group. The earlier removal of the catheter in the HoLEP group may be attributed to reduced bleeding and improved hemostasis during the postoperative period. In fact, in some cases, the catheter could be safely removed on the evening of the surgery day. Consistent with the results, previous studies have also reported significantly shorter hospitalization and catheterization times in patients undergoing HoLEP. Spinos et al further emphasized that same-day catheter removal is a feasible, safe, and effective approach in selected patients.^18^^,^^19^

When the groups were evaluated in terms of surgical safety, no significant difference was observed in complication rates. The most common complication in both groups was transient macroscopic hematuria, classified as Clavien–Dindo grade 1. At grade 2, urinary tract infection was the most frequent complication and was successfully managed with oral antibiotic therapy.

When hemoglobin parameters were evaluated, an average decrease of approximately 1 g/dL was observed at 24 hours postoperatively; however, this reduction was not considered clinically significant and was similar between the 2 groups. In contrast, Aizezi et al reported that hemoglobin reduction may be more clinically relevant, particularly in patients with large prostate volumes and advanced age, and that HoLEP was superior to TURP in terms of postoperative hemoglobin loss in their study.^20^

The findings indicate that both methods are effective and safe; nevertheless, HoLEP demonstrated certain advantages over B-TURP, including a greater amount and percentage of resected tissue, a more pronounced increase in Qmax, and shorter hospital stay and catheterization duration. These results suggest that HoLEP may be superior to B-TURP, which is traditionally considered the standard method not only for large prostates but also for small prostates, in several aspects, and therefore, even in patients with small prostates, both clinicians and patients should consider HoLEP when selecting the most appropriate surgical treatment for BPH.

This study has several limitations. First, its retrospective cross-sectional design limits causal inference and carries an inherent risk of selection bias. The choice between B-TURP and HoLEP was primarily based on surgeon preference, device availability, and institutional practice during the study period rather than on predefined selection criteria. Therefore, unmeasured confounding factors may have influenced treatment allocation and outcomes. Second, pressure-flow urodynamic studies, which would have allowed for a more precise differentiation, were not performed; although IPSS and uroflowmetry parameters provide general information about detrusor activity, they do not allow for distinguishing between normal and low activity. Third, although the follow-up period was sufficient to evaluate early functional and perioperative outcomes, it was limited to 6 weeks and therefore does not allow assessment of mid- or long-term functional durability, reoperation rates, or late complications. Longer-term follow-up studies are needed to better clarify the sustained efficacy and safety of both surgical techniques. Future multicenter studies with larger patient populations are expected to provide more robust and generalizable results.

## Conclusion

In the present study, both B-TURP and HoLEP were found to be safe and effective in small-volume prostates. HoLEP, however, demonstrated certain advantages over B-TURP, including a greater amount of resected tissue, a more pronounced increase in Qmax, and shorter hospital stay and catheterization duration. These findings suggest that HoLEP may offer potential advantages over B-TURP in selected perioperative and functional outcomes, not only in large prostate volumes but also in small prostates. In clinical practice, the choice of surgical technique should be individualized based on patient expectations, surgeon experience, and institutional resources.

## Figures and Tables

**Table 1.** Comparison of Demographic, Perioperative, and Postoperative Variables Between the HoLEP and TURP Groups

Variables	**HoLEP (n = 50)**	**B-TURP (n = 50)**	**Total (n = 100)**	*P*
Age (years)	65 (33)	68.5 (34)	66 (34)	.828
Comorbidity Hypertension (%) Diabetes mellitus (%)	14 (28) 12 (24)	18 (36) 16 (32)	32 (32) 28 (28)	.521 .504
PSA (ng/mL)	4.1 ± 5	5.1 ± 6.4	4.6 ± 5.7	.418
PV (cc)	45.3 ± 5.4	45.4 ± 4.8	45.3 ± 5.1	.875
Surgery time (minutes)	60 (95)	50 (55)	55 (95)	.03*
Spend saline (l)	16.8 ± 8.9	13.3 ± 5.3	15.1 ± 7.5	.02*
Resected tissue (g)	28.5 (22)	20 (28)	26 (29)	<.001*
Resected tissue (%)	63.3 (33.2)	45.6 (67.5)	60 (67.5)	<.001*
Length of stay (d)	1.6 ± 0.6	2.6 ± 0.76	2.1 ± 0.87	<.001*
Catheterization (d)	1.6 ± 0.6	2.8 ± 0.8	2.2 ± 0.94	<.001*
ICOQ-SF	0 (10)	0 (16)	0 (16)	.472
Complications (%)	5 (10)	7 (14)	12 (12)	.342
Clavien–Dindo classification				
Grade 1 (%)	4 (80)	5 (71.4)	9 (75)	
Grade 2 (%)	1 (20)	2 (28.6)	3 (25)	
Grade 3	0	0	0	.999
Grade 4	0	0	0	
Grade 5	0	0	0	

HoLEP, group that underwent holmium laser enucleation of the prostate; B-TURP, group that underwent bipolar transurethral resection of the prostate.

Quantitative variables that met parametric assumptions were expressed as mean ± standard deviation (SD), whereas those that did not were presented as median (interquartile range). Categorical variables are expressed as number (percentage). Values marked with an asterisk (*) are statistically significant.

ICIQ-SF, International Consultation on Incontinence Questionnaire-Short Form; PSA, prostate-spesific antigen; PV, prostate volume.

**Table 2.** Within-Group Preoperative and Postoperative Comparisons and Between-Group Comparisons of Measurement Differences (Postoperative–Preoperative) in the HoLEP and TURP Groups

Variables	HOLEP (n = 50)			B-TURP (n=50)			Comparison of Differences
	Pre-op	Post-op	*p*	Pre-op	Post-op	*p*	
Qmax(mL/s)	6.7 (11)	21.5 (43.1)	<.001*	6.5 (12)	19.8(39.4)	<.001*	.025*
IPSS	18 (17)	4 (18)	<.001*	18 (28)	5 (18)	<.001*	.243
IIEF-5	12 (20)	11(15)	.393	10 (17)	10.5 (15)	.195	.212
PVR(mL)	100 (40)	42.5 (10)	<.001*	100 (40)	45 (10)	<.001*	.591
Hemoglobin(g/dL)	14.7 (9)	13.8 (6.9)	<.001*	14.5 (8.5)	13.6 (7.5)	<.001*	.494

HoLEP, group that underwent holmium laser enucleation of the prostate; B-TURP, group that underwent bipolar transurethral resection of the prostate.

Variables are presented as median (interquartile range).

Values marked with an asterisk (*) are statistically significant.

IIEF-5, International Index of Erectile Function-5 items; IPSS, Internatıonal Prostate Symptom Index; PVR, postvoidal residual urine; Qmax, peak urinary flow rate.

## Data Availability

The data that support the findings of this study are available on request from the corresponding author.
